# Microporation is a valuable transfection method for efficient gene delivery into human umbilical cord blood-derived mesenchymal stem cells

**DOI:** 10.1186/1472-6750-10-38

**Published:** 2010-05-13

**Authors:** Jung Yeon Lim, Sun Hwa Park, Chang Hyun Jeong, Ji Hyeon Oh, Seong Muk Kim, Chung Hun Ryu, Soon A Park, Jae Geun Ahn, Wonil Oh, Sin-Soo Jeun, Jong Wook Chang

**Affiliations:** 1 Department of Biomedical Science, College of Medicine, The Catholic University of Korea, Seoul, Korea; 2 Department of Neurosurgery, Seoul St. Mary's Hospital, The Catholic University of Korea, Seoul, Korea; 3 Department of Neurosurgery, St. Paul's Hospital, The Catholic University of Korea, Seoul, Korea; 4 Medipost Biomedical Research Institute, MEDIPOST Co., Ltd., Seoul, Korea

## Abstract

**Background:**

Mesenchymal stem cells (MSCs) are an attractive source of adult stem cells for therapeutic application in clinical study. Genetic modification of MSCs with beneficial genes makes them more effective for therapeutic use. However, it is difficult to transduce genes into MSCs by common transfection methods, especially nonviral methods. In this study, we applied microporation technology as a novel electroporation technique to introduce enhanced green fluorescent protein (EGFP) and brain-derived neurotropfic factor (BDNF) plasmid DNA into human umbilical cord blood-derived MSCs (hUCB-MSCs) with significant efficiency, and investigated the stem cell potentiality of engineered MSCs through their phenotypes, proliferative capacity, ability to differentiate into multiple lineages, and migration ability towards malignant glioma cells.

**Results:**

Using microporation with EGFP as a reporter gene, hUCB-MSCs were transfected with higher efficiency (83%) and only minimal cell damage than when conventional liposome-based reagent (<20%) or established electroporation methods were used (30-40%). More importantly, microporation did not affect the immunophenotype of hUCB-MSCs, their proliferation activity, ability to differentiate into mesodermal and ectodermal lineages, or migration ability towards cancer cells. In addition, the BDNF gene could be successfully transfected into hUCB-MSCs, and BDNF expression remained fairly constant for the first 2 weeks *in vitro *and *in vivo*. Moreover, microporation of BDNF gene into hUCB-MSCs promoted their *in vitro *differentiation into neural cells.

**Conclusion:**

Taken together, the present data demonstrates the value of microporation as an efficient means of transfection of MSCs without changing their multiple properties. Gene delivery by microporation may enhance the feasibility of transgenic stem cell therapy.

## Background

Stem cells are highly attractive and valuable candidates for biomedical applications including the development of cell and gene therapy. Of the various stem cells, mesenchymal stem cells (MSCs) show a particular potential for clinical use because of their high proliferative capacity, ability to differentiate into multiple lineages [1-3], and ability to migrate into injured organs [4,5] and cancers [6,7]. Moreover, MSCs are not immunogenic, and so do not elicit a proliferative response of allogeneic lymphocytes *in vitro *[8]. Therefore, MSCs have become a major focus of research for potential therapeutic applications for various diseases.

In recent years, efforts have been made to improve the therapeutic efficacy of MSCs through combination approaches using MSCs and genes. Additionally, a new therapeutic strategy has been developed that uses MSCs for the targeted delivery and local production of biologic agents in tumors [6,9]. Viruses are commonly used as vehicles to deliver transgenes into stem cells that can be available to effectively infect dividing or nondividing cells. Integrating virus, including retrovirus or lentivirus, can insert their viral DNA into the host genomic DNA, which allows for stable genetic modification for the life of the host cells. So, they are very efficient for long-term gene expression [10-12]. Alternatively, nonintegrating viruses, including adenovirus or herpes saimirii virus, are preferentially used to obtain the expression of a therapeutic gene for a short time, although these viruses are less efficient at transferring genes into cells [13]. Overall, virus systems permit efficient gene delivery into cells; however, they have safety concerns that are critical when considering clinical applications [14]. Furthermore, their use sometimes causes significant changes in the characteristics of genetically modified cells [15].

To overcome these problems, nonviral methods are receiving increasing attention because they have several potential advantages over recombinant viruses. They are noninfectious, relatively nonimmunogenic, have low acute toxicity, can accommodate large DNA plasmids, and can be produced simply on a large scale [16]. There are various types of nonviral systems used for gene transfer, such as the liposome-based method [17], electroporation [18], and calcium phosphate techniques [19]. Electroporation, which permeabilizes the cell membrane by an electric pulse, has been widely used [20-22]. However, these methods are limited by their low gene transfer efficiency compared with viruses and their transient gene expression [16]. In addition, high cell mortality is still a problem in electroporation. Low levels of gene transfer continue to be a major obstacle in the use of nonviral systems because gene delivery and efficient gene transfer are prerequisites for the development of MSC therapy using various beneficial genes in clinical trials.

In contrast to conventional electroporation methods, microporation is a unique electroporation technology that uses a pipette tip as an electroporation space and a capillary type of electric chamber instead of a cuvette, which counteracts the harmful effects of cuvette-based electroporation gene transfer techniques such as pH variation, increasing in temperature, and metal ion generation.

The present study reports a nonviral, high-efficiency method of transfecting human umbilical cord blood-derived MSCs (hUCB-MSCs) using the new electroporation-based gene transfer technique of microporation. Microporation was utilized to introduce enhanced green fluorescent protein (EGFP) as a reporter gene and a plasmid expressing the gene of interest, brain-derived neurotrophic factor (BDNF), into hUCB-MSCs with significant efficiency, and investigated the stem cell potentiality of engineered hUCB-MSCs through their phenotypes, proliferative capacity, ability to differentiate into multiple lineages, and migration ability towards malignant glioma cells.

## Results

### Microporation of hUCB-MSCs Induces High Transient Transfection Efficiency

**Figure 1:**
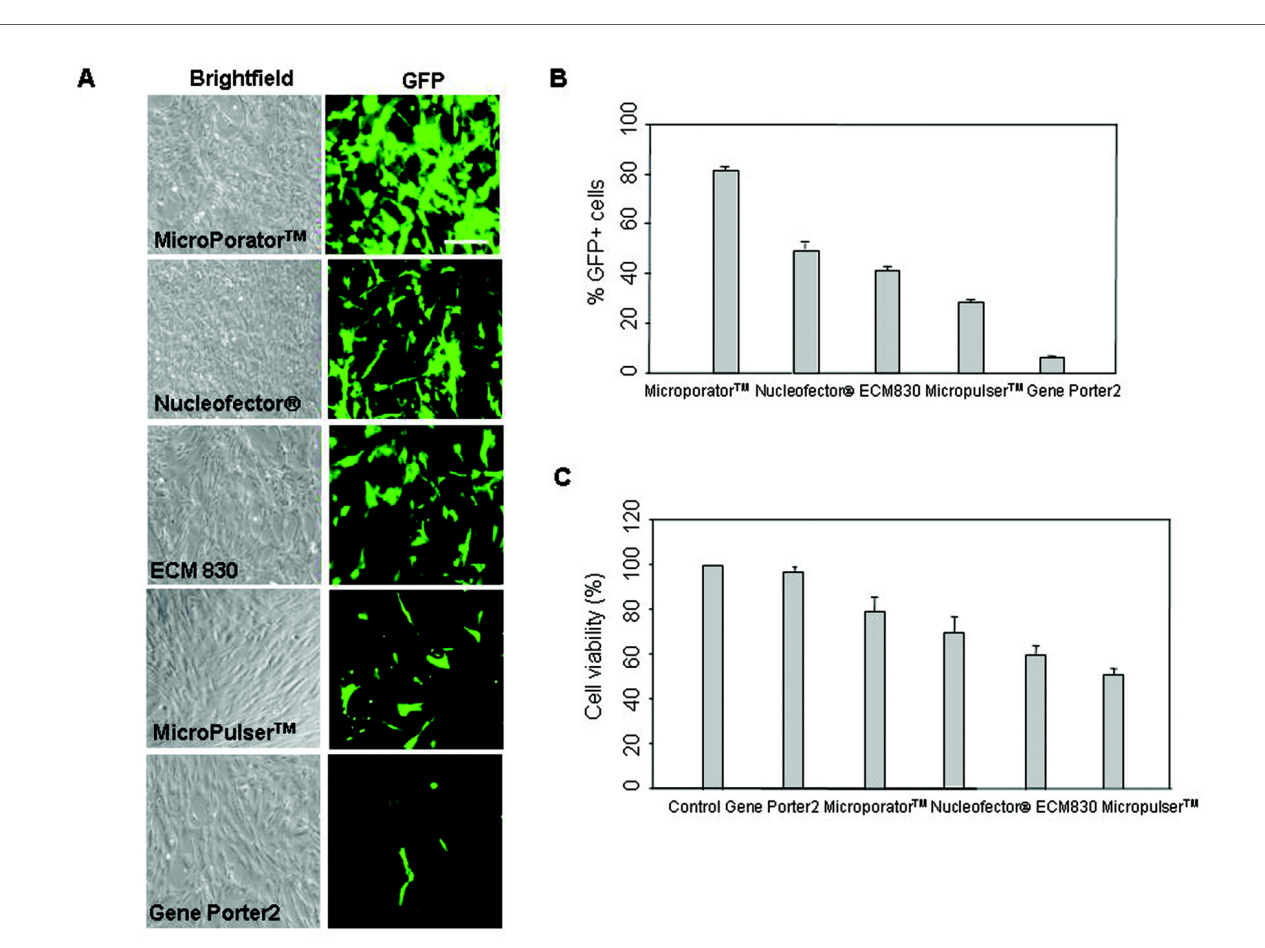
**Transfection efficiency of hUCB-MSCs**. (A) pEGFP-N1 vector was transfected into hUCB-MSCs by the established electroporators Nucleofector^®^, ECM 830, MicroPulser™, and a novel electroporator, MicroPorator™, or by a liposome-based reagent, Gene Porter2. After 24 hr, EGFP expression was analyzed using phase contrast and fluorescence microscopy. (B) Transient expression of EGFP was assessed by FACS analysis. (C) After transfection, cells were cultured for additional 48 hr and analyzed for viability by the MTS assay. The data are expressed as the mean ± SEM; n = 3. Scale bar = 200 μm.

To investigate the gene transfection efficiency of the microporation technique, hUCB-MSCs were transfected with EGFP-N1 plasmid (pEGFP-N1) by various transfection methods including liposome-based reagent, established electroporations, and microporation. For electroporation, 2 × 10^5 ^cells were resuspended in phosphate buffered saline (PBS) or resuspension buffer, mixed with 2 μg DNA and electroporated as described previously [23,24]. For microporation, 2 μg DNA was mixed with resuspension buffer. Then, 2 × 10^5 ^cells were electroporated with a preoptimized square pulse condition (1600 V, 20 ms, 1 pulse). Figure 1A showed the brightfield and green fluorescence images of cells. In addition, the percentage of EGFP-positive cells 24 hr after transfection was determined by fluorescence-activated cell sorting (FACS) analysis (Figure 1B). The percentage of EGFP-positive cells was 30%-50% for the established electroporators, Nucleofector^®^, ECM 830 or MicroPulser™. By comparison, the MicroPorator™ microporation device achieved a transfection efficiency of 83% under the conditions tested. In addition, microporation-based transfection of hUCB-MSCs showed a higher cell survival rate compared with other systems (Figure 1C). Although Gene Porter2-based transfection of hUCB-MSCs demonstrated a high cell survival rate, EGFP expression was too low (< 20%).

**Figure 2:**
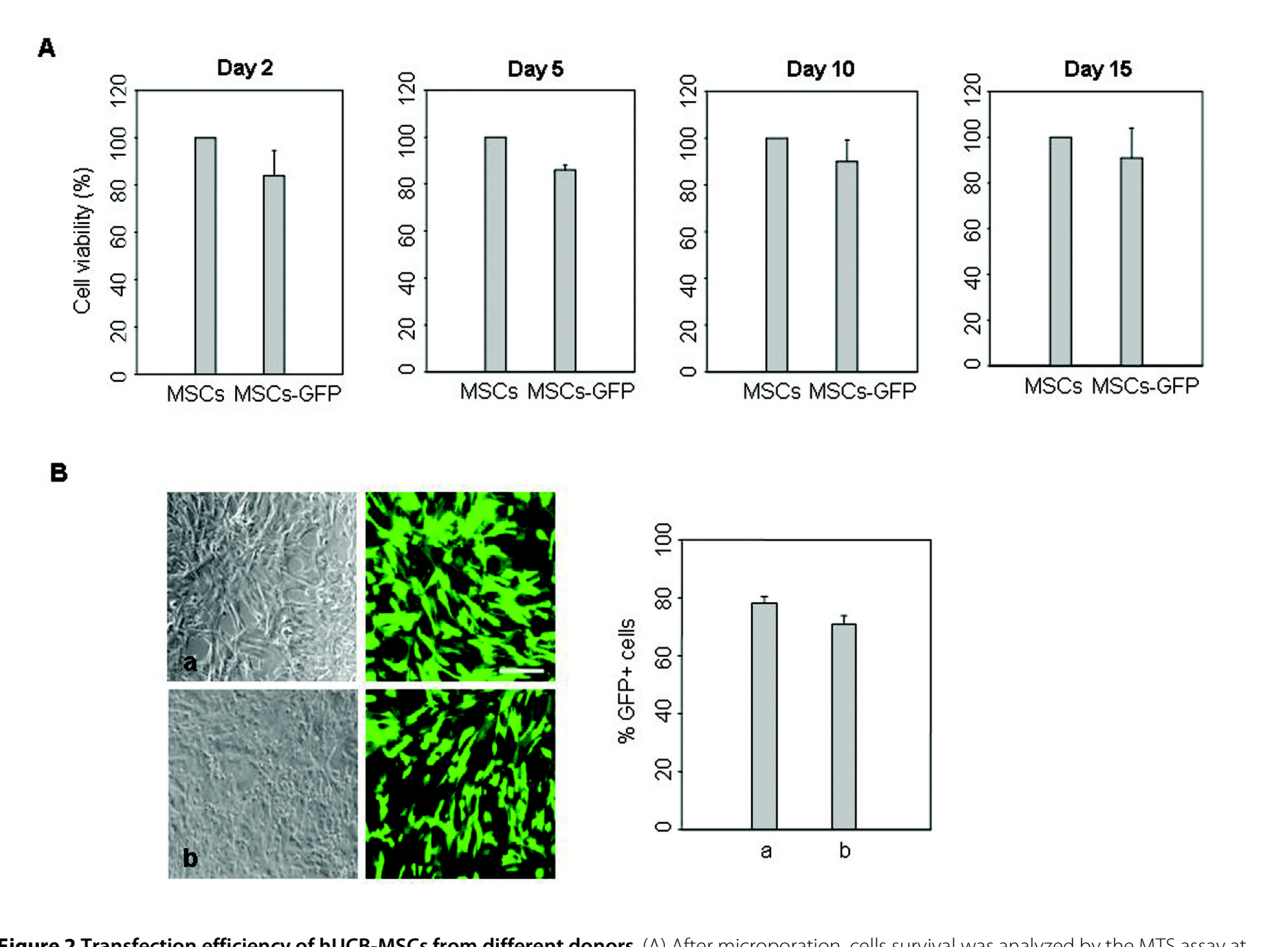
**Transfection efficiency of hUCB-MSCs from different donors**. (A) After microporation, cells survival was analyzed by the MTS assay at different time points under standard culture conditions (a-MEM containing 10% FBS and subculturing at 90% confluency). (B) pEGFP-N1 vector was transfected into different hUCB-MSCs from different donors by microporation. After 24 hr, the expression of EGFP was analyzed using phase contrast and fluorescence microscopy. Transient expression of EGFP was assessed by FACS analysis. The data are expressed as the mean ± SEM; n = 3. Scale bar = 200 μm.

We further examined the survival of microporated cells during 15 days culture of actively dividing cells. Cell viability was about 80% 2 days after microporation, and viability was sustained for more than 15 days (Figure 2A). To increase the application performance of microporation, transfection efficacies were examined in different hUCB-MSCs from different donors. Similar transfection efficiencies were obtained (Figure 2B).

**Figure 3:**
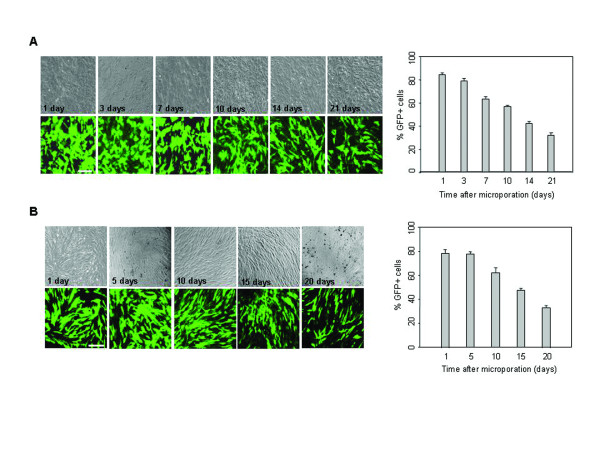
**Time-course analysis of EGFP expression in microporated hUCB-MSCs**. (A) pEGFP-N1 (2 μg) was transfected into hUCB-MSCs by microporation. The expression of EGFP was analyzed using phase contrast and fluorescence microscopy at different time points after transfection. Persistence of EGFP expression in hUCB-MSCs was analyzed by FACS analysis under conditions of restrained cell division (a-MEM containing 2% FBS and no passaging). EGFP expression was assessed at different time points after transfection. (B) Persistence of EGFP expression in microporated hUCB-MSCs was analyzed by FACS analysis under standard culture conditions. EGFP expression was assessed at different time points after transfection. The data are expressed as the mean ± SEM; n = 3. Scale bar = 200 μm.

**Figure 4:**
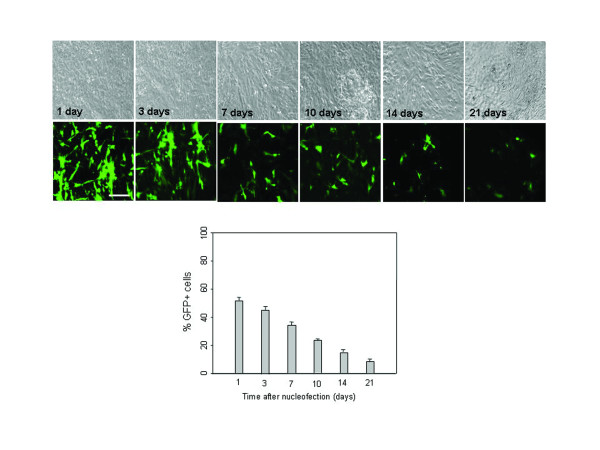
**Time-course analysis of EGFP expression in nucleofected hUCB-MSCs**. pEGFP-N1 (2 μg) was transfected into hUCB-MSCs by nucleofection. The expression of EGFP was analyzed using phase contrast and fluorescence microscopy at different time points after transfection. Persistence of EGFP expression in hUCB-MSCs was analyzed by FACS analysis under conditions of restrained cell division (a-MEM containing 2% FBS and no passaging). EGFP expression was assessed at different time points after transfection. The data are expressed as the mean ± SEM; n = 3. Scale bar = 200 μm.

To assess the stability of the microporation-based transfection, the time course and the level of EGFP expression were determined over a period of 3 weeks under conditions of restrained cell division (a-minimal essential medium (MEM) containing 2% fetal bovine serum (FBS) and no passaging). Fluorescence images and FACS analysis at different time points after transfection revealed that EGFP expression was sustained at high levels (>60%) for the first 10 days and began to decline after 2 weeks (Figure 3A). We also examined the EGFP expression level in actively dividing cells under standard culture conditions (a-MEM containing 10% FBS and subculturing at 90% confluency). Similarly, EGFP expression was sustained at high levels (>60%) for the first 10 days and began to decline after 15 days (Figure 3B). By comparison, EGFP expression was decreased markedly 10 days after nucleofection-based transfection, and its expression level was weak (Figure 4). These results indicate that transfection by microporation is an efficient way of obtaining successful gene delivery into MSCs.

### Multiple Stem Cell Traits of EGFP-expressing hUCB-MSCs by Microporation

**Figure 5:**
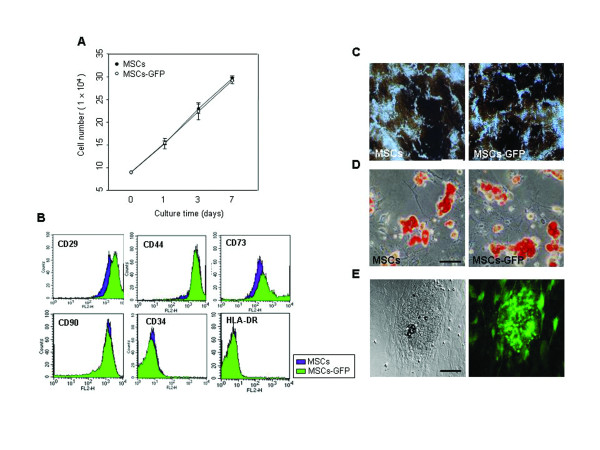
**Multiple stem cell traits of EGFP-expressing hUCB-MSCs**. (A) hUCB-MSCs were transfected with the 2 μg of pEGFP-N1 by microporation. After microporation, cells were cultured for additional 24 hr and analyzed with the trypan blue exclusion assay from 1-7 days. (B) Cells were labeled with antibodies against the indicated antigens and they were then analyzed by flow cytometry. hUCB-MSCs were transfected with the 2 μg of pEGFP-N1. After transfection, cells were allowed to differentiate under the specific induction medium. The accumulation of calcium-containing mineral deposits in the extracellular matrix and lipidic vacuoles were revealed by the (C) von Kossa staining method and (D) Oil Red O solution after 3 weeks. (E) EGFP expression was maintained in hUCB-MSCs during the osteoinductive conditions. The data are expressed as the mean ± SEM; n = 3. Scale bar (C) = 200 μm. Scale bar (D,E) = 100 μm.

To investigate the stem cell potentiality of hUCB-MSCs according to transfection by Microporatot™, cell proliferative capacity was evaluated by a trypan blue exclusion assay. Both non-transfected hUCB-MSCs and hUCB-MSCs transfected with pEGFP-N1 grew in a similar fashion in a time-dependent manner (Figure 5A). The *in vitro *culture of cells appeared to be unaffected by transfection using the microporation.

To further study stem cell potentiality, the expression of MSC-related cell surface antigens was evaluated by flow cytometry (Figure 5B). Like hUCB-MSCs, transfected hUCB-MSCs were strongly positive for the CD29, CD44, CD73, and CD90 markers. However, both cells stained negatively for HLA-class II (HLA-DR) and the hematopoietic marker CD34.

The ability to differentiate into a variety of cell types is an important characteristic of MSCs [2,3]. To evaluate the influence of microporation on multilineage differentiation of hUCB-MSCs, hUCB-MSCs and hUCB-MSCs transfected with pEGFP-N1 by microporation were induced into osteogenic and adipogenic differentiation. Both cells showed similar differentiation into the osteogenic and adipogenic lineages when grown in culture with the appropriate differentiation medium. Osteogenic differentiation was determined by von Kossa staining (Figure 5C). Adipogenic differentiation was apparent in the cells by cellular accumulation of lipid-rich vacuoles that were stained with Oil Red O (Figure 5D). EGFP expression was maintained in hUCB-MSCs during the osteoinductive conditions (Figure 5E).

**Figure 6:**
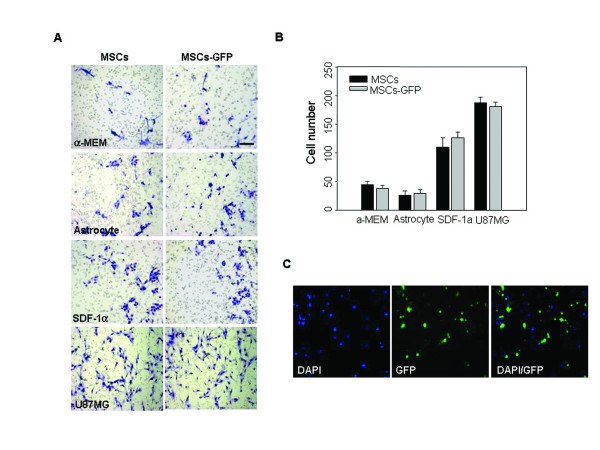
**Migration capacity of EGFP-expressing hUCB-MSCs**. (A) hUCB-MSCs were transfected with the 2 μg of pEGFP-N1. After transfection, the migration response of hUCB-MSCs to conditioned medium from U-87MG cells or normal astrocytes was determined using a Transwell plate. Cell migration was compared and evaluated after staining by taking photographs and (B) counting cells that had migrated. Serum free medium was used as a negative control and SDF-1 was used as a positive control. The number of cells that had migrated to the lower side of the filter was counted in five or six different microscopic fields under a light microscope. (C) The expression of EGFP was observed in migrated hUCB-MSCs. Nuclei were counterstained with DAPI (blue). Experiments were performed in triplicate. Scale bar = 200 μm.

Migration capacity towards cancer cells is an important characteristic of MSCs. Recent studies have indicated that MSCs exhibit potent tropism for malignant glioma cells [7,9]. To test this in hUCB-MSCs transfected with pEGFP-N1 by microporation, *in vitro *migration assays using Transwell plates were conducted. Conditioned medium (CM) from normal human astrocytes was used as a control to mimic the normal brain milieu. Only a few cells migrated toward serum-free medium (SFM) and CM from normal human astrocytes, whereas the migration of cells was significantly stimulated by CM from the human glioma cell line U-87MG compared with CM from astrocytes. We also confirmed that transfected hUCB-MSCs migrated towards CM from U-87MG cells in a similar pattern to hUCB-MSCs (Figure 6). In addition, the expression of EGFP was observed in migrated hUCB-MSCs. Thus, the migratory ability of hUCB-MSCs was not affected by microporation.

### Characterization of BDNF-expressing hUCB-MSCs by Microporation

To investigate the potential for application of microporated hUCB-MSCs for cell therapy, the gene of interest harbored in a BDNF expression plasmid (pEGFP-BDNF) was transfected into hUCB-MSCs and the transgene expression and its effects for promoting *in vitro *differentiation of hUCB-MSCs into neural cells were examined. BDNF regulates the survival, development, and function of neurons, and is important for differentiation of neural precursor cells [25,26]. Our previous report demonstrated that BDNF stimulates *in vitro *differentiation of MSCs into neural cells [27]. In addition, MSCs genetically modified with the BDNF gene effectively promote axonal regeneration in transected adult rat spinal cord [28]. Therefore, if hUCB-MSCs could be successfully modified with BDNF gene by microporation, it would be a valuable cell source for the treatment of neurological disease.

**Figure 7:**
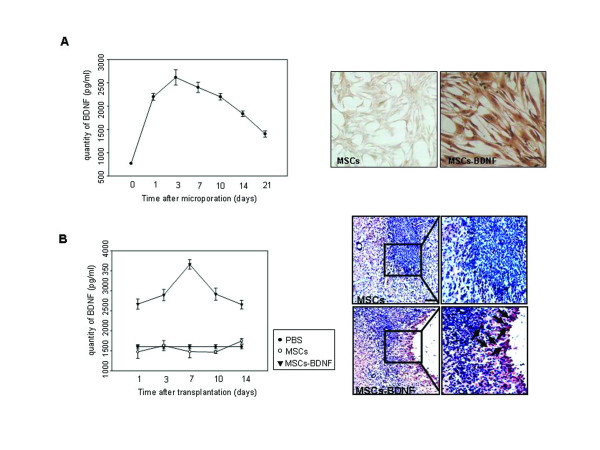
**Time-course analysis of BDNF expressions in microporated hUCB-MSCs**. (A, left panel) pEGFP-BDNF (2 μg) was transfected into hUCB-MSCs by microporation. The concentration of secreted BDNF was determined using an ELISA assay under conditions of expanded cells (a-MEM containing 10% FBS). (A, right panel) Cells were stained for BDNF 3 days after transfection. (B, left panel) For the quantification of BDNF levels and longevity of BDNF expression *in vivo*, brain tissues were homogenized at 1, 3, 7, 10, and 14 days (*n *= 3/each group) after treatment and then assessed by ELISA. (B, right panel) Tissues were stained for BDNF 3 days after transplantation. Nuclei were counterstained with Hematoxylin (blue). The data are expressed as the mean ± SEM; n = 3. Scale bar = 200 μm.

Enzyme-linked immunosorbant assay (ELISA) analysis at different time points after transfection revealed that the concentration of secreted BDNF *in vitro *remained fairly constant for 14 days and then began to decline in transfected hUCB-MSCs (Figure 7A, left panel). Additionally, immunocytochemical analysis showed strong staining for BDNF in the transfected cells (Figure 7A, right panel). The levels of BDNF protein and its longevity *in vivo *were quantitatively measured. ELISA analysis on different days after transplantation of cells into rat brain revealed that the BDNF protein expressed in brain tissues was detectable on day 1, peaked on day 7, and persisted for two weeks. In control groups, expression levels of BDNF were low throughout the experimental period (Figure 7B, left panel). In addition, immunohistochemical analysis 3 days after transplantation showed strong staining for BDNF in the injection site or areas around the injection site of BDNF-expressing hUCB-MSCs (Figure 7B, right panel).

**Figure 8:**
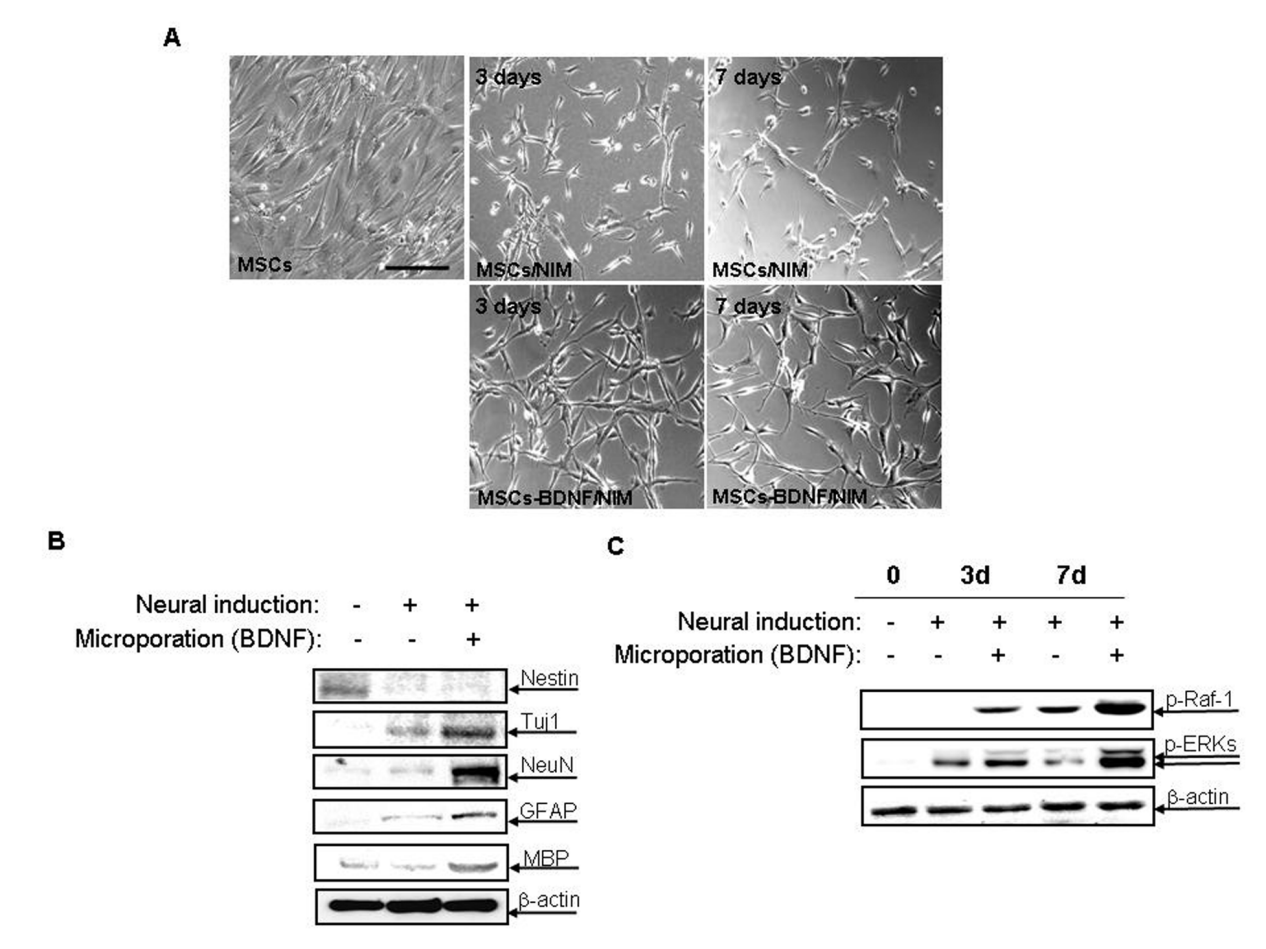
**Neurogenic characteristics of BDNF-expressing hUCB-MSCs**. (A) phase-contrast microscopy analysis of hUCB-MSCs after 3 and 7 days incubation in NIM, with or without microporation-based pEGFP-BDNF transfection (2 μg). (B) Western blotting of hUCB-MSCs before and 3 days of incubation in NIM with or without pEGFP-BDNF transfection. The cell lysates were then subjected to SDS-polyacrylamide gel electrophoresis and Western blot analysis with anti-nestin, anti-Tuj-1, anti-NeuN, anti-GFAP, and anti-MBP antibodies. (C) Western blotting of hUCB-MSCs before and 3 and 7 days of incubation in NIM with or without pEGFP-BDNF transfection. The cell lysates were then subjected to SDS-polyacrylamide gel electrophoresis and Western blot analysis with anti-phospho ERK, -phospho -Raf-1 antibodies. Equal amounts of total protein were confirmed by using an anti-β-actin antibody. The results shown here are from one of two independent experiments. Scale bar = 100 μm

To investigate the effect of BDNF in promoting *in vitro *differentiation of hUCB-MSCs into neural cells, cell morphology was observed when hUCB-MSCs were treated with neural induction medium (NIM), with or without transfection of BDNF expression plasmid by microporation Non-transfected or transfected hUCB-MSCs were incubated overnight in preinduction medium containing bFGF. Next, they were transferred to NIM, and then maintained for up to 7 days. hUCB-MSCs transfected with BDNF expression plasmid progressively assumed neuronal morphological characteristics. The cytoplasm in the flat hUCB-MSCs retracted toward the nucleus, resembling a typical neuronal soma, and the process became thinner because of continuous shrinkage of the cell body. The morphological changes progressed to yield network-like structures and many long processes. However, these morphological changes were weak for the non-transfected hUCB-MSCs, and only a few cells showed pyramidal or spherical cell bodies with multiple processes (Figure 8A). In addition to their neural morphological differentiation, the effects of BDNF were investigated by Western blot analysis. In the proliferating medium, MSCs highly expressed the intermediate filament protein nestin. But, the expression of nestin decreased dramatically after induction. MSCs were negative for several neural proteins including the differentiating neuron marker Tuj-1, the mature neuron marker NeuN, GFAP typical of astrocytes, and the oligodendrocyte marker MBP. Conversely, expression of Tuj-1 and NeuN increased significantly after neurogenic stimulation in BDNF-transfected hUCB-MSCs. In addition, expression of GFAP and MBP increased after induction in transfected cells. However, these increases were marginal for those non-transfected cells treated with NIM (Figure 8B).

Next, the neural differentiation-related molecules in the hUCB-MSCs and BDNF-transfected hUCB-MSCs were examined after neural induction. Mitogen-activated protein kinases are expressed abundantly in the central nervous system, and extracellular signal-regulated kinases (ERKs) participate in various activity-dependent processes, including neuronal maturation, survival, and synaptic functions. The phosphorylation levels of Raf-1 and ERKs were dramatically increased in BDNF-transfected hUCB-MSCs at 7 days of induction (Figure 8C).

## Discussion

MSCs therapy using therapeutic transgene holds the potential as a powerful means of disease treatment. For example, MSCs genetically modified with the BDNF gene effectively promote axonal regeneration in transected adult rat spinal cord [28] and also protect against injury in a cerebral ischemia model in adult rats [29]. MSCs genetically modified with bone morphogenic protein-2 (BMP-2) gene show good results in treating bone defects, and engineering MSCs with BMP-2 has recently been the focus for research into the treatment of a variety of bone defects [30,31].

Many advances have been achieved in the field of gene transfer; however, some problems such as poor efficiency and maintenance of cell characteristics remain. Transfection of hard-to-transfect cells, including primary neuron cells, primary blood cells, and stem cells through common methods such as the use of a liposome-based reagent or electroporation is problematic because of low transfection efficiency and low cell survival rates.

Genetically modified cells should maintain the transgene wherever and whenever they are treated, as well as their cell characteristics. If MSCs can be successfully modified, more potential cells will be available for use in treatment. Therefore, technology and methodology that enables efficient gene transfer is needed.

In this study, we obtained successful transfection efficiency with only minimal cell damage by the microporation-based transfection with the EGFP reporter plasmid in hUCB-MSCs. Moreover, a plasmid expressing the gene of interest, BDNF, could be successfully transfected into hUCB-MSCs, and *in vitro *and *in vivo *experiments showed that BDNF expression remained fairly constant for the first 2 weeks (Figures 1, 2, 3, 4 and 7). These results show that gene delivery by microporaton is a safe and efficient means for prolonged transgene expression.

Recently, Wang et al. [32] demonstrated that microporation allows a highly efficient transfection of human adipose tissue-derived stem cells, without impairing their stem cell properties. Their results echo our data that transfection by microporation is an efficient way of obtaining successful gene delivery into hUCB-MSCs.

More importantly, microporation of BDNF gene into hUCB-MSCs presently promoted *in vitro *neural characteristics (Figure 8). MSCs can differentiate into neurons *in vitro *under special experimental conditions [33] and *in vivo *after transplantation into the brain and spinal cord [34,35]. In addition, transplanted MSCs promoted functional improvement in a spinal cord injury rat model [35]. Our previous results demonstrated that BDNF stimulates *in vitro *differentiation of MSCs into neural cells [27]. Furthermore, engineered MSCs with the BDNF gene effectively promote neural differentiation and also protect against injury in transected spinal cord or ischemia animal models [27,28,35]. Therefore, microporation-based successful genetic modification of hUCB-MSCs with BDNF makes them potential candidates for the treatment of neurological diseases.

Additionally, microporation-mediated transfection of pEGFP-N1 into hUCB-MSCs and EGFP overexpression did not affect the multiple potential of stem cells. This effect was demonstrated by cell phenotype characterization, proliferative activity, ability to differentiate into multiple lineages, and potent migration ability for malignant glioma cells (Figures 5 and 6). Maintenance of these multiple properties of modified stem cells is critical for improving their therapeutic potentials. Otherwise, stem cells may not be effective in a combination therapy of stem cells and therapeutic genes. Therefore, questions regarding the efficacy of gene transfection as well as safety and maintenance of multiple stem cell traits after gene modification need to be answered in greater detail for the effective utilization of stem cells in treating human diseases.

This study has established a reliable and efficient method for introducing a transgene into stem cells. Transfection by the microporation method led to 83% successful expression of transgene in hUCB-MSCs. In addition, multiple stem cell traits of hUCB-MSCs were unaffected by transfection. Successful genetic modification of stem cells requires achieving efficient DNA transfer into cells and maintaining their multiple cell characteristics. Therefore, transfection by the microporation is an efficient way of obtaining successful gene delivery into MSCs and gene delivery by microporation may enhance the feasibility of transgenic stem cell therapy.

## Conclusion

Successful transfection efficiency was obtained with only minimal cell damage by the microporation-based transfection with the EGFP reporter plasmid in hUCB-MSCs without changing their multiple properties. Moreover, a plasmid expressing the gene of interest, BDNF, could be successfully transfected into hUCB-MSCs. More importantly, microporation of BDNF gene into hUCB-MSCs promoted their *in vitro *neural characteristics. Therefore, transfection by the microporation is an efficient way of obtaining successful gene delivery into MSCs and gene delivery by microporation may enhance the feasibility of transgenic stem cell therapy.

## Methods

### Plasmid Construction

An EGFP expression vector, pEGFP-N1, was purchased from Clontech BD Bioscience (Palo Alto, CA, USA). BDNF cDNA was obtained using the Marathon cDNA Amplification system (Clontech) and cloned in the TOPO TA Cloning vector (Invitrogen, Carlsbad, CA, USA) and the sequence was verified. Plasmid encoding human BDNF was generated by polymerase chain reaction (PCR) cloning into pEGFP-N1 using *Kpn*I (5" primers) and *Not*I (3" primers) restriction sites, which was designated pEGFP-BDNF.

### hUCB-MSCs and Transfection

Human UCB samples were obtained with consent from the mothers and were separated and maintained in accordance with techniques as previously described [36]. Cells were transfected with pEGFP-N1 or pEGFP-BDNF by liposome-based reagent, Gene Porter2 (Gene Therapy System, San Diego, CA, USA), established electroporators; Nucleofector^® ^(Amaxa, Cologne, Germany), ECM 830 (BTX Harvard Apparatus, Holliston, MA, USA), MicroPulser™ (Bio-Rad, Huston, TX, USA) and a novel electroporator (MicroPorator™, Digital Bio, Seoul, Korea).

For electroporation, cells were trypsinized and resuspended in PBS for electroporation with the ECM 830 or in resuspension buffer for electroporation with the MicroPulser™ and Nucleofector^®^. Then 2 × 10^5 ^cells were used for each electroporation. Briefly, 2 μg DNA was mixed in PBS and electroporated as described previously [23,24]. For microporation, 2 μg DNA was mixed with 10 μl resuspension buffer. Then, 2 × 10^5 ^cells were electroporated with a pre-optimized square pulse condition (1600 V, 20 ms, 1 pulse). Electroporated cells were incubated in 500 μl α-MEM (Invitrogen) supplemented with 10% FBS (Invitrogen) without antibiotics for 24 hr, and then the medium was replaced with a medium containing antibiotics. To assay transient and sustained expression, the hUCB-MSCs were isolated by incubation in 0.25% trypsin and 1 mM EDTA (Invitrogen) and assayed by a FACSCalibur flow cytometer (Becton Dickinson, Franklin Lakes, NJ, USA) 24 hr after transfection for EGFP-expressing cells. The results were analyzed by CellQuest software (Becton Dickinson).

### Assessment of Cell Viability

hUCB-MSCs were transfected with pEGFP-N1 by various transfection. Medium was removed 24 hr after transfection, and replaced with medium containing antibiotics. Cells were analyzed for viability by CellTiter 96^® ^aqueous non-radioactive cell proliferation assay (Promega, Madison, WI, USA) at different time points after transfection. The assay tests cellular viability and mitochondrial function. Briefly, after transfection, MTS solution was added to each culture plate and plates were incubated at 37°C for 3 hr. The absorbance of the formazan product, which is considered to be directly proportional to the number of living cells in the culture, was measured at 490 nm using an ELISA plate reader.

### Animals and Cell Transplantation

Adult male SD rats (6-8 weeks old; Charles River Laboratories, Wilmington, MA, USA) were used in accordance with institutional guidelines under the approved protocols. Cells were transfected with pEGFP-BDNF and maintained further for 48 hr in α-MEM containing 10% FBS. For the intracranial xenografts of hUCB-MSCs, animals were anesthetized with an intraperitoneal injection of ketamine/xylazine and 3 × 10^5 ^cells were stereotactically transplanted into the right frontal lobe (2.6 mm lateral and 1.2 mm anterior to bregma, at 5.0 mm depth from the skull base) via a Hamilton syringe (Hamilton Company, Reno, NV, USA) using a microinfusion pump (Harvard Apparatus, Holliston, MA, USA).

### ELISA for Expressed BDNF

To investigate the persistence of transgene expression *in vitro*, hUCB-MSCs were transfected with pEGFP-BDNF by MicroPorator™. Culture supernatants were harvested, fresh medium (α-MEM containing 10% FBS) was added, and secreted BDNF was assessed at various time intervals. To investigate the persistence of BDNF expression *in vivo*, brain tissues (2 mm segment centered on the injection site) were harvested and lysed in a RIPA buffer at 1, 3, 7, 10, and 14 days after treatment of cells. BDNF protein secreted into the culture supernatants or brain tissues was analyzed by ELISA assay kits (R&D Systems, Minneapolis, MN, USA).

### Immunophenotyping of hUCB-MSCs

To analyze the cell surface expression of typical marker proteins in hUCB-MSCs, cells were labeled with the following anti-human antibodies: CD29-PE, CD34-PE, CD44-PE, CD73-PE, CD90-PE, and HLA-DR-PE (BD Biosciences). Ten thousand cells were measured using a FACSCalibur flow cytometer (Becton Dickinson) and the results were analyzed with CellQuest software (Becton Dickinson).

### Multidifferentiation of Cultured hUCB-MSCs

Differentiation to adipogenic and osteogenic lineages was induced according to previously described procedures [2]. After 2-3 weeks of culture, differentiated cells were fixed with 3% formaldehyde. Adipocytes were detected by staining the lipid droplets in the cell using 0.3% Oil red-O staining for 10 min. Osteocytes were detected by calcium phosphate deposits after von Kossa staining. In brief, cells were fixed with ethanol and stained with 5% silver nitrate for 1 hr. After rinsing with distilled water, the cells were incubated in 5% sodium thiosulfate for 2 min to allow precipitation of insoluble black silver granules around calcium phosphate.

### Immunocytochemistry

After transfection, cells were fixed with 4% paraformaldehyde for 10 min and processed for immunocytochemistry to identify neural marker-positive cells. Nonspecific antibody reactions were blocked with 5% horse serum for 1 hr at room temperature (RT). Next, the fixed cells were incubated overnight at 4°C with primary antibodies directed against BDNF (Chemicon, Temecula, CA, USA). After three washes, cells were incubated with biotinylated secondary antibodies (Vector Laboratories, Burlingame, CA, USA) for 1 hr at RT, followed by 1 hr of incubation in avidin-biotinylated peroxidase complex (Vector Elite Kit, Vector Laboratories) at RT. Diaminobenzidine (0.05%) with nickel chloride (0.04%) was used as the chromagen, and reactions were sustained for 1-6 min at RT. The fixed cells were then coverslipped with Fluoromount G (Southern Biotechnology Associates, Birmingham, AL, UK).

### Immunohistochemistry

Rat brains were perfused with PBS followed by 4% paraformaldehyde under deep anesthesia at 3 days after transplantation of hUCB-MSCs or transfected hUCB-MSCs. The excised brains were postfixed overnight and then equilibrated in PBS containing 30% sucrose for 2 days. Fixed brains were embedded, snap frozen in liquid nitrogen, and stored at -70°C until use. Tissues were cryosectioned (16 μm) and then stained with primary antibodies for anti-BDNF (Chemicon). After three washes, tissues were incubated with biotinylated secondary antibodies (Vector Laboratories) for 1 hr at RT, followed by 1 hr of incubation in avidin-biotinylated peroxidase complex (Vector Elite Kit, Vector Laboratories) at RT. Vector NovaRED (Vector Laboratories) was used for visualizing the immunoreaction products, and reactions were sustained for 1-6 min at RT. Nuclei were counterstained with Hematoxylin.

### In vitro Migration Assay

The migratory ability of hUCB-MSCs was determined using Transwell plates (Corning Costar, Cambridge, MA, USA) that were 6.5 mm in diameter with 8 μm pore filters. 1 × 10^6 ^of cells were incubated in 5 ml serum free MEM for U-87MG or DMEM for astrocytes for 48 hr, and the resulting conditioned media were used as chemoattractants. hUCB-MSCs or hUCB-MSCs transfected with pEGFP-N1 (2 × 10^4^) were suspended in 100 μl serum free medium (SFM) containing 0.1% bovine serum albumin (Sigma) and seeded into the upper well, and 600 μl of conditioned medium (CM) was placed in the lower well of the Transwell plate. Following incubation for 5 hr at 37°C, cells that had not migrated from the upper side of the filters were scraped off with a cotton swab, and filters were stained with the three-step stain set (Diff-Quik; Sysmex, Kobe, Japan). The number of cells that had migrated to the lower side of the filter was counted under a light microscope (×200). Experiments were performed in triplicate.

### Neural Induction Procedure

Neural induction was performed in accordance with the procedure described by Lim et al. [27]. In brief, cells were plated in α-MEM containing 10% FBS at 4000 cells/cm^2^. Twenty-four hours before induction, the medium was replaced with a preinduction medium composed of α-MEM, 10% FBS and 10 ng/ml of basic fibroblast growth factor (bFGF, Invitrogen). The cells were induced by replacing the pretreatment medium with neural induction medium (NIM), consisting of 100 μM butylated hydroxyanisole (Sigma), 2% dimethylsulfoxide (Sigma), 25 mM KCl, 5 U/ml heparin (Sigma), 20 ng/ml bFGF, 5 μg/ml insulin (Sigma), 100 μg/ml transferring (Sigma), 20 nM progesterone (Sigma), 100 μM putrescine (Sigma), 30 nM sodium selenite (Sigma), and 0.5 μM all-trans-retinoic acid (Sigma). After induction, the cells were maintained in NIM for up to 7 days.

### Western Blot Analysis

Antibodies were obtained from commercial sources: BDNF, Nestin, βIII tubulin, NeuN, and MBP antibodies from Chemicon; GFAP antibody from Dako; p-ERKs, and p-Raf-1 antibodies from New England Biolabs (Ipswich, MA). For the Western blot analysis, the cells were rinsed with PBS and subsequently lysed for 30 min on ice in RIPA-B buffer (0.5% Nonidet P-40, 20 mM Tris, pH 8.0, 50 mM NaCl, 50 mM NaF, 100 μM Na3 VO4, 1 mM DTT, and 50 μg/ml PMSF). The insoluble material was removed by centrifugation at 12,000 rpm for 20 min at 48 C. Next, the supernatant was subjected to SDS-PAGE, and the Western blot analysis was then performed. The blots were blocked in PBS with 5% skim milk and 0.05% Tween 20, incubated with the appropriate antibodies and subsequently incubated with the secondary antibodies conjugated with horseradish peroxidase. Next, the blots were assayed using an enhanced chemiluminescence detection system (Amersham Biosciences, Piscataway NJ, USA).

## Authors' contributions

JYL conceived the method, generated the experimental data, and wrote the first draft of the manuscript. SSJ participated in the evaluation of the data. Cell transplantation and *in vitro *migration experiments were performed by CHJ and SMK with help and advice from JAJ and CHR. Microporation experiments were performed by JHO and SHP. Immunostaining experiments were performed JGA and SAP. hUCB-MSCs were prepared by JWC and WIO. All authors read and approved the final manuscript.
